# Primary Human Cardiomyocytes and Cardiofibroblasts Treated with Sera from Myocarditis Patients Exhibit an Increased Iron Demand and Complex Changes in the Gene Expression

**DOI:** 10.3390/cells10040818

**Published:** 2021-04-06

**Authors:** Kamil A. Kobak, Paweł Franczuk, Justyna Schubert, Magdalena Dzięgała, Monika Kasztura, Michał Tkaczyszyn, Marcin Drozd, Aneta Kosiorek, Liliana Kiczak, Jacek Bania, Piotr Ponikowski, Ewa A. Jankowska

**Affiliations:** 1 Laboratory for Applied Research on Cardiovascular System, Department of Heart Diseases, Wroclaw Medical University, 50-556 Wroclaw, Poland; kamkobak@gmail.com (K.A.K.); pawelfranczuk3@gmail.com (P.F.); magdalena.stugiewicz@gmail.com (M.D.); michal.tkaczyszyn@umed.wroc.pl (M.T.); marcin.drozd@umed.wroc.pl (M.D.); 2 Centre for Heart Diseases, University Hospital, 50-556 Wroclaw, Poland; anetakosiorek7@gmail.com (A.K.); piotr.ponikowski@umed.wroc.pl (P.P.); 3 Department of Food Hygiene and Consumer Health Protection, Wroclaw University of Environmental and Life Sciences, 50-375 Wroclaw, Poland; justyna.schubert@upwr.edu.pl (J.S.); monika.kasztura@upwr.edu.pl (M.K.); jacek.bania@upwr.edu.pl (J.B.); 4 Department of Heart Diseases, Wroclaw Medical University, 50-556 Wroclaw, Poland; 5 Department of Biochemistry and Molecular Biology, Wroclaw University of Environmental and Life Sciences, 50-375 Wroclaw, Poland; liliana.kiczak@upwr.edu.pl

**Keywords:** myocarditis, iron metabolism, iron deficiency

## Abstract

Cardiac fibroblasts and cardiomyocytes are the main cells involved in the pathophysiology of myocarditis (MCD). These cells are especially sensitive to changes in iron homeostasis, which is extremely important for the optimal maintenance of crucial cellular processes. However, the exact role of iron status in the pathophysiology of MCD remains unknown. We cultured primary human cardiomyocytes (hCM) and cardiofibroblasts (hCF) with sera from acute MCD patients and healthy controls to mimic the effects of systemic inflammation on these cells. Next, we performed an initial small-scale (*n* = 3 per group) RNA sequencing experiment to investigate the global cellular response to the exposure on sera. In both cell lines, transcriptomic data analysis revealed many alterations in gene expression, which are related to disturbed canonical pathways and the progression of cardiac diseases. Moreover, hCM exhibited changes in the iron homeostasis pathway. To further investigate these alterations in sera-treated cells, we performed a larger-scale (*n* = 10 for controls, *n* = 18 for MCD) follow-up study and evaluated the expression of genes involved in iron metabolism. In both cell lines, we demonstrated an increased expression of transferrin receptor 1 (TFR1) and ferritin in MCD serum-treated cells as compared to controls, suggesting increased iron demand. Furthermore, we related TFR1 expression with the clinical profile of patients and showed that greater iron demand in sera-treated cells was associated with higher inflammation score (interleukin 6 (IL-6), C-reactive protein (CRP)) and advanced neurohormonal activation (NT-proBNP) in patients. Collectively, our data suggest that the malfunctioning of cardiomyocytes and cardiofibroblasts in the course of MCD might be related to alterations in the iron homeostasis.

## 1. Introduction

Myocarditis (MCD) is defined as an inflammation of cardiac muscle [1]. Some of the patients with MCD recover spontaneously, but others develop post-myocarditis non-ischemic cardiomyopathy, which often progresses to the need for mechanical circulatory support or even heart transplantation [2,3,4]. MCD is predominantly induced by viruses but also by other infectious agents such as bacteria, protozoa, and fungi. Viral infection may affect the heart directly by the infiltration of myocardium, or it may indirectly induce cardiac injury by triggering a systemic cytokine storm or a cellular immune response by molecular mimicry [5]. However, the molecular mechanisms behind the cardiac response to the systemic inflammation in the course of myocarditis remain largely unexplored. Investigation of these mechanisms might help understanding why some patients develop the aforementioned inflammatory cardiomyopathy with a poor prognosis and reveal efficient therapeutic options.

Iron has been shown to be an important modulator of the myocardial stress response [6]. In recent years, the relation between disrupted iron homeostasis and its consequences for heart has been deeply investigated [7]. Both iron deficiency (ID) and iron overload are associated with structural and functional abnormalities within cardiac cells and heart tissue [8,9,10,11,12,13]. As a cofactor of numerous proteins and enzymes, iron stands at the crossroads of many essential biological processes such as cellular energy metabolism, oxygen transport and storage, and importantly—immune response, anti-infectious mechanisms, and processes curing inflamed tissues [14,15,16,17,18,19]. Thus, the optimal availability of this essential micronutrient is critical for the survival of all types of cells [14,17]. Moreover, cells with high mitogenic potential and high energy demand are particularly sensitive to depleted iron supply, excessive iron load, or abnormal iron utilization [14,15,17,20]. The aforementioned issues could be of particular importance in the context of MCD, as the major cells involved in the pathophysiology of this disease are immune cells, cardiomyocytes, and cardiofibroblasts, and their functioning strongly relies on iron availability.

The goal of this study was to investigate the cellular response to the systemic inflammation in the course of MCD using an in vitro model. Although the role of iron status in MCD and its potential progression to non-ischemic cardiomyopathy is not well understood, clinical characterization of the investigated group of MCD patients showed decreased serum iron levels along with increased serum ferritin. These clinical premises suggest altered systemic iron homeostasis as a significant modulator of the complex pathophysiology of MCD. Thus, special emphasis was focused on iron metabolism and its possible link to cardiac remodeling in MCD.

To mimic the clinical conditions of MCD-related systemic inflammation in our experimental model, we cultured primary human cardiomyocytes (hCM) and cardiofibroblasts (hCF) with the addition of sera collected from patients: (1) in the acute phase of myocarditis, (2) after 6 weeks of clinical recovery, and (3) healthy controls. First to validate the correctness of our model, we performed a small-scale RNA sequencing experiment to observe global effects of the treatment with sera from MCD patients on the gene expression patterns in hCM and hCF. Next, in a larger-scale experiment, we examined the expression of genes and proteins involved in intracellular iron metabolism i.e., transferrin receptor 1 (TFR1)—cellular iron importer, whose expression level is an indicator of cellular iron demand [21,22,23]; ferritin (light and heavy chains; FTL and FTH), which plays a key role in iron storage but also is an acute phase protein [24]. Finally, we compared the results of our in vitro study with a clinical profile of patients whose sera were used to treat the cells. We hypothesize that cardiac cells exposed to the sera from myocarditis patients will exhibit pathological changes in the gene expression along with disrupted iron homeostasis.

## 2. Materials and Methods

### 2.1. Experimental Schedule

The cells were maintained according to the manufacturer’s protocol. Primary human cardiomyocytes (hCM; PromoCell, Heidelberg, Germany) were precultured in Myocyte Growth Medium supplemented with the recommended Supplemented Mix (PromoCell) for 60 days in order to induce differentiation toward myotube-like and branch-like structures. Primary human cardiofibroblasts (hCF, PromoCell) were precultured in Fibroblast Growth Medium supplemented with the recommended Supplemented Mix (PromoCell) and passaged 5 times before the experiment. Cells were cultured on Nunc™ Cell-Culture 6 or 24-well plates (Thermo Fisher Scientific; Waltham, Massachusetts, USA). For passaging, both cell lines were treated with DetachKit (PromoCell).

**Figure 1:**
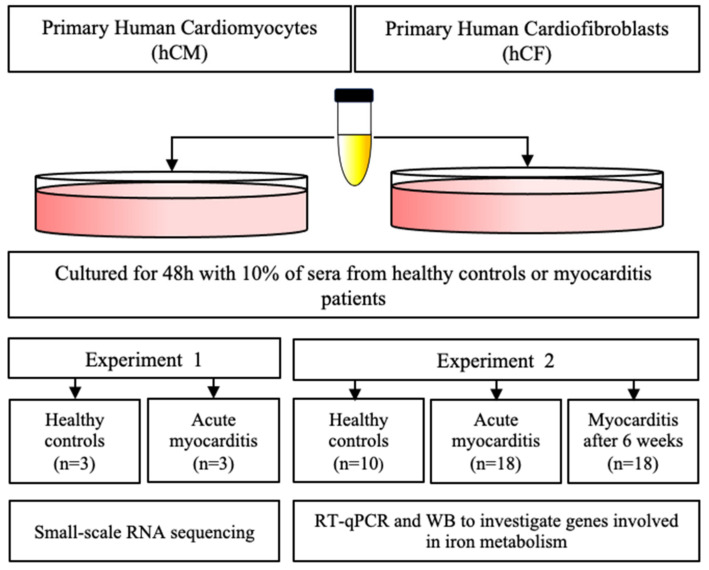
Schematic representation of experimental design and study protocol.

For experiments, hCM and hCF were cultured for 48 h with 10% of sera from patients instead of Supplemented Mix. Sera were sterilized by filtrating on cellulose acetate 0.22 μm centrifuge tube filters (Corning^®^ Costar^®^ Spin-X^®^, Sigma-Aldrich; Merck KGaA, Darmstadt, Germany). Two series of experiments were performed (Figure 1)—initial small-scale RNA sequencing study (*n* = 3 per group of healthy controls and acute myocarditis) and a follow-up study with a larger number of patients (*n* = 10 for controls and *n* = 18 for myocarditis patients at two time points). After 48 h exposure to patients’ sera, cells were washed, collected and lysed either with Trizol (for further usage in transcriptome experiments; Thermo Fisher Scientific; Waltham, MA, USA) or RLT buffer (for RT-qPCR and Western blotting (WB); Qiagen, Hilden, Germany). Cell culture supernatants collected from each series of experiments were centrifuged at 15,800× *g* for 10 min and frozen at −80 °C for several weeks.

### 2.2. Patients

A total of 18 consecutive male patients hospitalized for acute myocarditis were prospectively enrolled during 2014–2019. Acute myocarditis was diagnosed based on the following criteria: (1) new onset symptoms suggestive of myocarditis (shortness of breath, effort intolerance, fatigue, palpitations, or chest pain), (2) elevated high sensitivity cardiac troponin I (hs-cTnI), (3) exclusion of obstructive coronary artery disease in coronary angiography or coronary computed tomographic angiography, (4) cardiac magnetic resonance image suggestive of myocarditis, and (5) age ≥ 18 years. We recruited 10 healthy adult volunteers among collogues and relatives for control group. The study protocol was approved by the local ethics committee (Bioethics Committee, Wroclaw Medical University), and a written informed consent at inclusion was obtained from all subjects. The study was conducted in accordance with the Helsinki Declaration.

The following assessments were performed during both hospitalization and control ambulatory visit 6 weeks after discharge from hospital. Left ventricular ejection fraction (LVEF, %) was assessed in cardiac magnetic resonance. Blood was withdrawn in the morning and collected into Vacutainer tubes with clot activator. Tubes were inverted five to six times to mix clot activator and blood and incubated in an upright position at room temperature for 30–40 min to allow clotting. Tubes were spun at 2000× *g* for 15 min without brake. Then, serum was removed, aliquoted, and stored at −80 °C until use. The plasma level of N-terminal pro-B-type natriuretic peptide (NT-proBNP; pg/mL) was measured using an immunoassay based on chemiluminescence with Dimension RxL system (Siemens, Munich, Germany). Serum level of high-sensitivity C-reactive protein (hs-CRP; mg/L) was assessed using immunonephelometry with BN II System (Siemens). The following blood biomarkers/parameters reflecting iron metabolism were measured directly (from fresh venous blood): serum ferritin (μg/L), iron (mg/dL), and total iron-binding capacity (TIBC, mg/dL). Transferrin saturation (TSAT) was calculated as the ratio of serum iron (mg/dL) and TIBC (mg/dL) multiplied by 100 and expressed as a percentage. Serum iron and TIBC were assessed using a substrate method with the Konelab Prime 60i system (Thermo Scientific). Serum ferritin was measured using an immunoassay based on electrochemiluminescence with the Elecsys 2010 system (Roche, Basel, Switzerland).

### 2.3. Cell Viability Tetrazolium Reduction Assay (MTS)

MTS assays were performed, according to the manufacturer’s protocol (CellTiter 96^®^ AQueous One Solution Cell Proliferation Assay; Promega Corporation, Madison, WI, USA). Briefly, hCM or hCF cells were seeded into each well of 96-well plates and were treated for 48 h with patients’ sera or PBS (control), as described above. A total of 20 µL CellTiter 96^®^ AQueous One Solution reagent was added to each well, and the absorbance at 490 nm was measured after 2 h incubation in 37 °C (KCjunior™; BioTek Instruments, Inc., Winooski, VT, USA). The viability of the untreated cells was treated as 100%.

### 2.4. RNA Extraction and Library Preparation for mRNA Sequencing

For mRNA sequencing, total RNA extraction from Trizol was performed according to the manufacturer’s instructions. RNA concentration was measured using Qubit^®^ RNA Assay Kit in Qubit^®^ 2.0 Flurometer (Life Technologies, Carlsbad, CA, USA). RNA integrity was assessed using the RNA Nano 6000 Assay Kit of the Bioanalyzer 2100 system (Agilent Technologies, Santa Clara, CA, USA).

A total amount of 1 μg RNA per sample was used as input material for the RNA sample preparations. mRNA from eukaryotic organisms was enriched using oligo (dT) beads from NEBNext^®^ Poly (A) mRNA Magnetic Isolation Module (NEB, USA). Subsequently, sequencing libraries were generated using a NEBNext Ultra II Directional RNA Library Prep Kit for Illumina^®^ (NEB, USA) following the manufacturer’s recommendations. Briefly, fragmentation was carried out using divalent cations under elevated temperature in NEBNext First Strand Synthesis Reaction Buffer (5X). First-strand cDNA was synthesized using random hexamer primer and M-MuLV Reverse Transcriptase (RNaseH). Second-strand cDNA synthesis was subsequently performed using DNA Polymerase I and RNase H. In the reaction buffer, dNTPs with dTTP were replaced by dUTP. Remaining overhangs were converted into blunt ends via exonuclease/polymerase activities. After the adenylation of 3′ ends of DNA fragments, NEBNext Adaptors with hairpin loop structure were ligated for hybridization. In order to select cDNA fragments of preferentially 250~300 bp in length, the library fragments were purified with AMPure XP beads (Beckman Coulter, Beverly, USA). Then, 3 μL USER Enzyme (NEB, USA) was used with size-selected, adaptor-ligated cDNA at 37 °C for 15 min followed by 5 min at 95 °C. Then, PCR was performed with Phusion High-Fidelity DNA polymerase, Universal PCR primers and Index (X) Primer. At last, products were purified (AMPure XP beads), and library quality was assessed using the Agilent High Sensitivity DNA Kit (Agilent Technologies) on the Agilent Bioanalyzer 2100 system (Agilent Technologies).

### 2.5. RNA Sequencing and Transcriptome Analysis

Strand-specific cDNA libraries were sequenced on an Illumina Novaseq6000 NGS sequencer with the following parameters: PE150, minimum of 40 million reads (40M), which gave at least 12 gigabases (12Gb) per sample of raw data. Sequencing was performed on 12 RNA samples: hCM and hCF cells treated with sera from representative healthy controls (*n* = 3 per each group); hCM and hCF cells treated with sera from representative acute myocarditis patients (*n* = 3 per each group). Raw data were aligned to the reference genome GRCh38 *Homo sapiens* (GRCh38; Ensembl, v. 97) using Hisat2 [25], v. 2.1.0. For transcript abundance, Cuffquant and Cuffmerge (v. 2.2.1) tools were used [26]. Out of the 226,658 transcripts identified in the database, 95,090 were filtered out due to the very low expression level (the average expression level was less than 0.001 FPKM, Fragments Per Kilobase Million). Significant changes in gene expression were determined by separated comparison of hCM and hCF to control, using the Student’s *t*-test. The false discovery rate (FDR) was estimated using the Benjamini–Hochberg method [27]. Protein-coding transcripts that changed significantly based on unadjusted *p*-value < 0.05 and fold change >2 in both directions were subjected to pathway analysis by Ingenuity Pathway Analysis software (IPA; Qiagen). Canonical molecular pathways, biological processes, and toxicity functions databases were used by IPA to test their association with differentially regulated genes.

### 2.6. Reverse Transcription-Quantitative Polymerase Chain Reaction (RT-qPCR)

For RT-qPCR, total RNA extraction was performed according to the manufacturer’s instructions (AllPrep DNA/RNA/Protein Mini Kit, Qiagen). For the synthesis of the first-strand cDNA, the SuperScript III First-Strand Synthesis System with oligo(dT)20 primer (Invitrogen, Carlsbad, CA, USA) was applied. PCR primers were supplied by BioRad (PrimePCR™, Bio-Rad Laboratories, Inc., Hercules, CA, USA) or designed with Beacon Designer Software (version 2.0, Bio-Rad Laboratories). Primer sequences and reference numbers are listed in Table 1. In order to prevent the amplification of genomic DNA, the primers were designed from specified exon–exon junctions of genes of interest. The selection of reference genes was carried out by means of geNorm analysis (geNorm kit, ge-SY-12; PrimerDesign, Ltd., Southampton, UK), as a result of which the combination of two reference gene, *UBC* and *ATP5B* (both Homo sapiens), was identified as the most stable reference value across all experimental conditions. RNA quality was determined using PrimePCR™ RNA Quality SYBR^®^ Green Assay (Bio-Rad Laboratories)—samples with unsatisfying quality were excluded from analysis. RT-qPCR was carried out using the CFX ConnectTM Real-Time PCR Detection System (Bio-Rad Laboratories) and SsoFastTM EvaGreen^®^ Supermix reagent (Bio-Rad Laboratories,). For each reaction, a melt curve analysis was performed in order to determine the specificity of PCR. The amplification efficiency was estimated by running serial dilutions of a template or provided by manufacturer. For the target amplicons, *UBC* and *ATP5B*, the relative expression was calculated using ∆∆Cq method in Bio-Rad CFX Manager (version 3.1, Bio-Rad Laboratories).

### 2.7. Western Blotting (WB)

hCM and hCF were homogenized in RLT buffer (Qiagen), and total protein isolation was performed according to the manufacturer’s instructions (AllPrep DNA/RNA/Protein Mini Kit, Qiagen). The protein concentration was estimated by bicinchoninic acid (BCA) assay [28]. In order to determine protein levels of TFR1, FTH, FTL, and actin (as a loading control), 10 μg of appropriate protein lysates were added on 26 well 4–20% Criterion™ TGX Stain-Free™ Protein Gels (Bio-Rad Laboratories). Proteins were electro-transferred on polyvinylidene fluoride (PVDF) membranes using a Trans-Blot^®^ Turbo™ System (Bio-Rad Laboratories) using an RTA Midi PVDF Transfer Kit (Bio-Rad Laboratories) according to the manufacturer’s instructions.

The membranes were blocked with 5% skimmed milk for 1 h and incubated with primary antibodies overnight (Table 2). After washing, membranes were incubated with secondary horseradish-conjugated anti-rabbit antibodies and developed with the Femto detection system (Pierce Biotechnology, Rockford, IL, USA). Western blots were visualized and analyzed using film and ChemiDoc XRS+ System (Bio-Rad Laboratories).

### 2.8. Statistical Analysis

Most continuous variables had a normal distribution and were expressed as a mean ± standard deviation of the mean. NT-proBNP, CRP, interleukin 6 (IL-6), soluble transferrin receptor (sTfR), and ferritin had a skewed distribution and were log-transformed (a natural logarithm, ln) before further inclusion in linear regression analyses. These variables were expressed as a median with an interquartile range. Differences in values between healthy controls and acute MCD patients were analyzed with unpaired Student’ *t*-test or Mann–Whitney test. Differences in values between 3 groups of sera-treated cells were analyzed with the one-way ordinary ANOVA test followed by Dunnett’s multiple comparisons test or Kruskal–Wallis test followed by Dunn’s multiple comparisons test. Spearman’s rank correlation coefficient (R) reflects the relationship between the protein expression of TFR1 in hCM or hCF and iron status, and other laboratory measurements in peripheral blood. *p* < 0.05 was used to indicate a statistically significant difference. Statistical analyses were performed using the GraphPad Prism Software (GraphPad Software Inc., San Diego, CA, USA).

## 3. Results

### 3.1. Cell Viability in hCM and hCF Treated with Patients’ Sera

Cell MTS assay did not show any relevant changes in hCM viability when treated with both myocarditis (acute phase and 6 weeks follow up) and control sera as compared with hCM cultured in dedicated media. In hCF, treatment with patients or control sera caused 25% lower viability of cells, independently from the type of serum donor (data not shown).

### 3.2. Transcriptome Profiling Indicates Alterations in the Gene Expression in hCM and hCF Treated with Acute Myocarditis Serum

**Figure 2:**
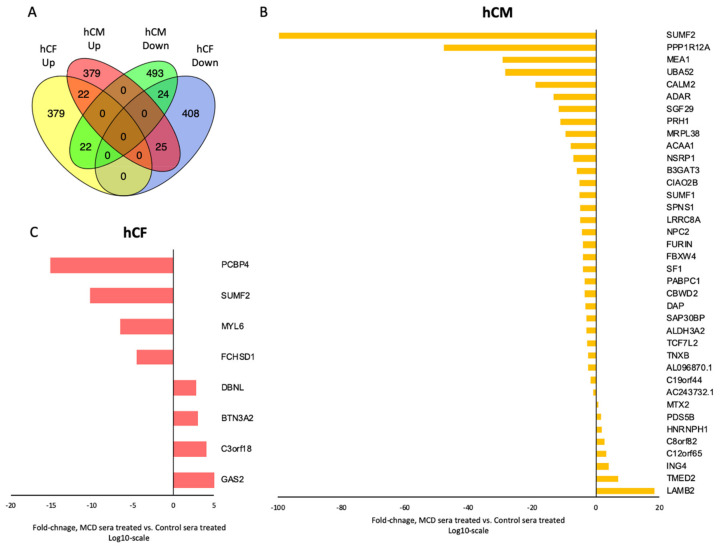
Venn diagram (**A**) of overlapped up- and downregulated genes detected between two cell types (hCF and hCM). Significantly changed genes in hCM (**B**) and hCF (**C**) treated with sera from acute MCD patients vs. healthy controls. Student’ *t*-test, *p*_FDR_ < 0.1, *n* = 3 per group. Abbreviations: hCM, human cardiomyocytes; hCF, human cardiofibroblasts.

To observe the complex effect of treatment and validate the correctness of model, next-generation RNA sequencing on a subset of sera-treated hCM and hCF (acute MCD vs. healthy control; *n* = 3 for each group) identified >226,000 transcripts. After the removal of low counts, 131,568 transcripts remained. RNAseq analysis revealed 2297 and 2017 transcripts that were significantly differentially expressed between MCD vs. healthy control serum-treated groups for hCM and for hCF, respectively (fold change >2; *p* < 0.05). Among them, for hCM, 436 unique genes were upregulated and 538 unique genes were downregulated. For hCF, the number of up- and downregulated unique genes was 424 and 457, respectively. Then, we compared the sets of altered genes between two cell types using Venn diagrams. The results displayed an overwhelmingly unique response of each cell type to the MCD serum treatment. Thus, only 22 common genes were upregulated and 24 common genes were downregulated in both cell types (Figure 2A).

To avoid false-positive scores, the results were corrected by FDR, revealing 38 genes that were significantly different in hCM (30 downregulated; 8 upregulated; Figure 2B) and 8 genes that were significantly different in hCF (4 downregulated; 4 upregulated; Figure 2C) compared to control.

**Figure 3:**
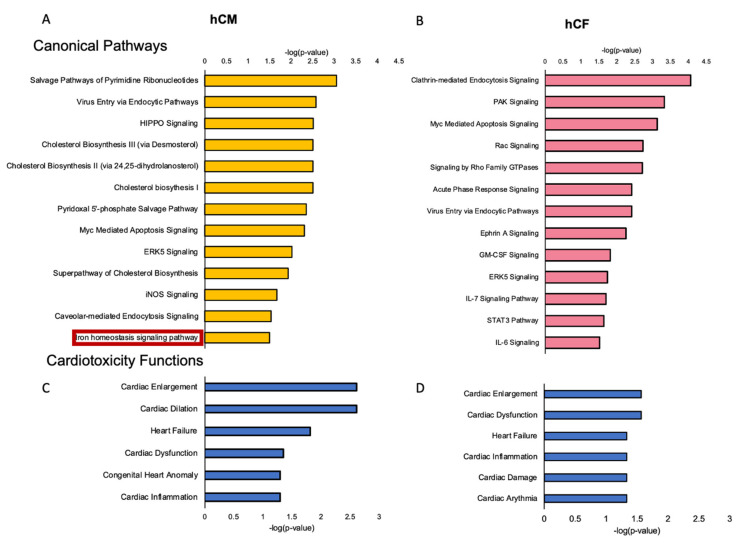
Selected hints of the significantly dysregulated canonical pathways in hCM (**A**) and hCF (**B**) treated with sera from acute MCD patients vs. healthy controls. Selected hints of the significantly dysregulated cardiotoxicity functions in hCM (**C**) and hCF (**D**) treated with sera from acute MCD patients vs. healthy controls. Identified with Ingenuity Pathway Analysis (IPA) using respectively 1138 and 1050 protein-coding transcripts that changed significantly. Fisher’s exact test, *p* < 0.05. Abbreviations: hCM, human cardiomyocytes; hCF, human cardiofibroblasts; ERK5, extracellular-signal-regulated kinase 5; iNOS, inducible nitric oxide synthase; PAK, p21-activated kinases; GM-CSF, granulocyte-macrophage colony-stimulating factor; IL-7, interleukin 7; STAT3, signal transducer and activator of transcription 3; IL-6, interleukin 6.

To further investigate the alterations in canonical molecular pathways and to better understand biological processes and cardiotoxicity functions caused by the treatment with MCD patients’ sera, Ingenuity Pathway Analysis (IPA) was performed. Significantly altered (unadjusted *p*-value < 0.05, and fold change >2) protein-coding genes were used for further pathway analysis (1043 and 956 detected transcripts for hCM and hCF, respectively). The analysis revealed multiple dysregulated canonical pathways associated with MCD serum treatment in both hCM and hCF as compared with cells treated with serum from healthy controls. Thus, among others, inflammation, endocytosis, virus entry via endocytic pathways, cholesterol biosynthesis, and apoptosis as well as iron homeostasis-related pathways were significantly dysregulated in hCM (*p* < 0.05; Figure 3A). In hCF, endocytosis and virus entry via endocytic pathways as well as apoptosis and inflammation (IL-6, IL-7) pathways were also altered. Interestingly, significant changes in the acute phase response pathways in MCD serum-treated were observed only in hCF (*p* < 0.05; Figure 3B). A list of all significantly altered pathways is given in Table S1A,B in the Supplementary Materials.

Detailed IPA analysis of cardiac-specific functions showed significant dysregulations in pathways related to cardiac enlargement, cardiac dilation, heart failure, cardiac dysfunction, and cardiac inflammation in both hCM and hCF (both *p* < 0.05; Figure 3C,D). In addition to changes in cardiac-specific functions, we observed significant alterations in pathways related to 22 and 24 different diseases in hCM and hCF, respectively (Supplementary Materials; Supplementary Figure S1A,B).

### 3.3. Increased Expression of TFR1 Indicates an Iron Depletion in Cells Treated with Sera from Myocarditis Patients

To further investigate the effect of serum treatment on cells in the context of iron metabolism, hCM and hCF were treated with sera from 18 patients in two timepoints (hospitalization for the acute phase of MCD and after 6 weeks of clinical recovery) and from 10 healthy controls (without any cardiovascular disease). The baseline characteristics of all examined patients are presented in Table 3.

**Figure 4:**
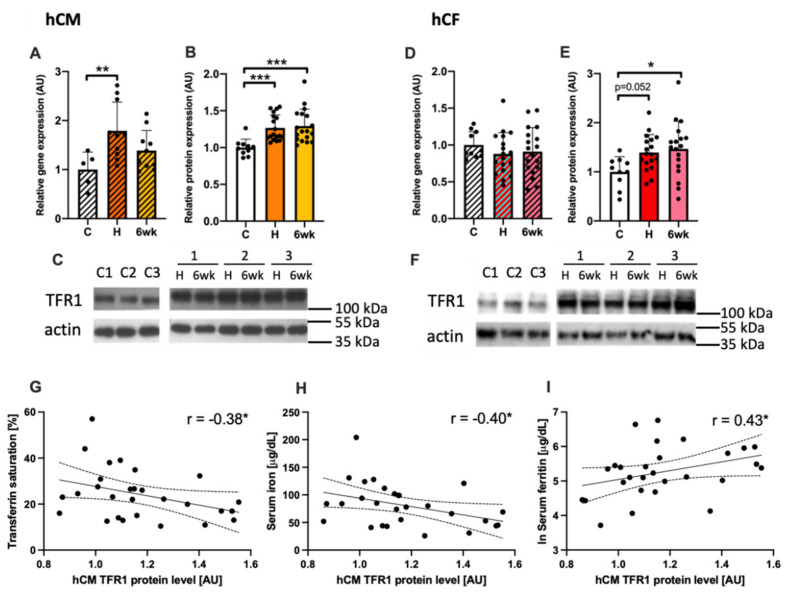
The expression of TFR1 in human cardiomyocytes (**A**–**C**) and in human cardiofibroblasts (**D**–**F**) treated with patients’ sera at the mRNA and protein level, together with representative immunoblots (**C**, **F**). ANNOVA test followed by Dunnett’s multiple comparisons test or Kruskal–Wallis test followed by Dunn’s multiple comparisons test (depending on data normality). *p < 0.05; ** *p* < 0.01; *** *p* < 0.001. The association between the protein level of TFR1 in human cardiac myocytes treated with patient sera and markers of iron status in patients’ peripheral blood i.e., transferrin saturation (**G**), serum iron (**H**), and serum ferritin (**I**). Gene and protein expressions were normalized to mean value in control group. Spearman’s rank correlation. * *p* < 0.05; ** *p* < 0.01; *** *p* < 0.001. Abbreviations: AU, arbitrary units; C, healthy controls, H, hospitalization (acute phase); 6wk, after 6 weeks of clinical recovery; TFR1, transferrin receptor 1; TSAT, transferrin saturation.

hCM exposed to sera from acute MCD patients showed a significant increase in an expression of TFR1 both at the mRNA (*p* < 0.01; Figure 4A) and protein level (*p* < 0.001, Figure 4B) in comparison to those treated with sera from healthy controls. This observation suggests an increased iron demand in cardiomyocytes cultured in myocarditis-serum treatment conditions. While in hCF treated with acute MCD sera, the overexpression of TFR1 was observed only at the protein level (*p* < 0.05 Figure 4E). Interestingly, the level of TFR1 protein was also increased when cells (both hCM and hCF) were treated with MCD patients’ sera collected after 6 weeks of clinical recovery, indicating that the iron depletive effect of sera from MCD patients on cells is continuous.

Additionally, the expression of TFR1 protein in hCM was correlated with systemic iron status in patients (controls + acute MCD) measured in peripheral blood i.e., transferrin saturation (TSAT, R = −0.38; *p* < 0.05; Figure 4G), serum iron (R = −0.41; *p* < 0.05; Figure 4H) and serum ferritin (R = 0.43; *p* < 0.05; Figure 4I).

### 3.4. Increased Expression of Ferritin in the Course of Myocarditis

**Figure 5:**
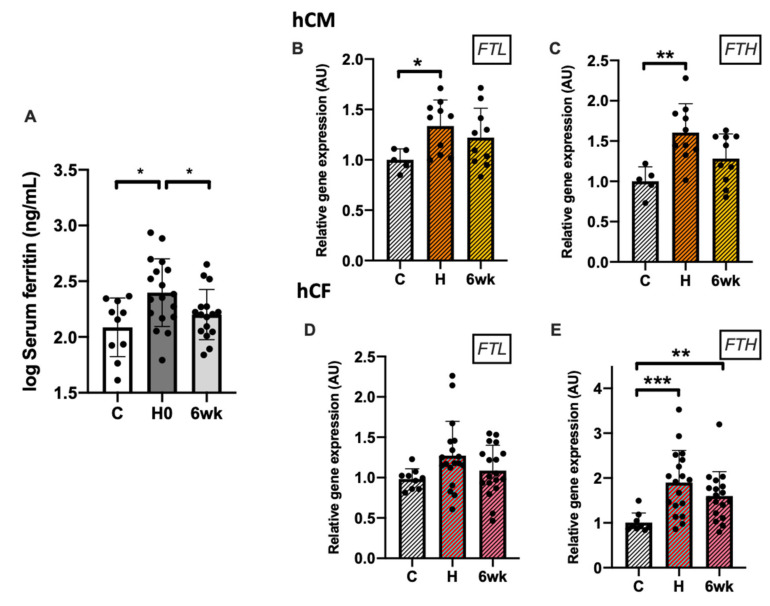
The levels of ferritin in patient sera (**A**) and the mRNA expression of *FTL* and *FTH* in human cardiac myocytes (**B**,**C**) and human cardiac fibroblasts (**D**,**E**) treated with patients’ sera. Gene expression was normalized to mean value in control group. ANOVA test followed by Dunnett’s multiple comparisons test or Kruskal–Wallis test followed by Dunn’s multiple comparisons test (depending on data normality). * *p* < 0.05; ** *p* < 0.01; *** *p* < 0.001. Abbreviations: U, arbitrary units; C, healthy controls, H, hospitalization (acute phase); 6wk, after 6 weeks of clinical recovery; FTL, ferritin light chain; FTH, ferritin heavy chain.

Serum ferritin level was higher in acute myocarditis patients as compared to healthy controls and got normalized after 6 weeks of clinical recovery (*p* < 0.05; Figure 5A). Both hCM and hCF treated with acute myocarditis patients’ sera exhibited an increase in *FTL* and *FTH* expression at the mRNA (all *p* < 0.05; Figure 5B–D) but not at the intracellular protein level (data not shown). Additionally, we measured the level of extracellular ferritin secreted to culture media where we also did not observe any significant differences (data not shown).

### 3.5. Disturbed Iron Metabolism in Cells Is Related to Inflammation and Outcome Parameters of Patients

**Figure 6:**
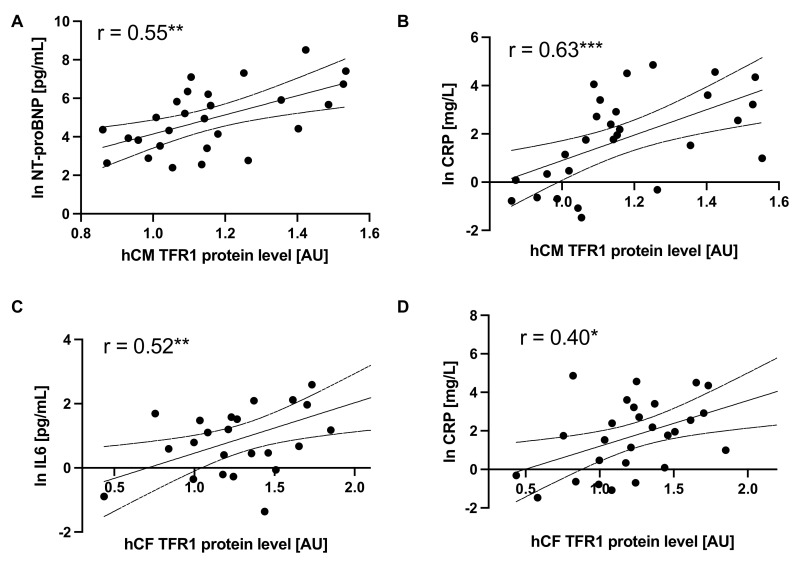
The association between the protein level of TFR1 in sera-treated hCM or hCF and serum levels of CRP (**A**,**B**), IL-6 (**C**) and NT-proBNP (**D**). Protein expression was normalized to mean value in control group. Spearman’s rank correlation. * *p* < 0.05; ** *p* < 0.01; *** *p* < 0.001. Abbreviations: CRP, C-reactive protein; NT-proBNP, N-terminal pro-B-type natriuretic peptide; IL6, interleukin 6.

Higher expression of TFR1 in both hCM and hCF treated with sera was associated with elevated levels of CRP in subjects (R = 0.63 and R = 0.49 respectively; Figure 6B,D). Moreover, in hCM, we observed a significant correlation between cellular TFR1 expression and the level of NT-proBNP (R = 0.55; *p* < 0.01; Figure 6A) in subjects’ sera. Additionally, in hCF, the higher expression of TfR1 protein was also correlated with the augmented level of IL-6 in subjects’ sera (R = 0.52; *p* < 0.01; Figure 6C). These data suggest that escalating iron demand in both cell lines increases with advanced neurohormonal activation in patients (NT-proBNP) and augmented inflammation (CRP, IL-6).

## 4. Discussion

In this study, we investigated the effects of sera collected from myocarditis patients on human cardiac myocytes and cardiofibroblasts. Treatment with sera from patients with various cardiac diseases has been shown to induce changes in many different processes in cultured cells. Recently, Garcia et al. showed that the treatment with single ventricle heart disease sera results in detrimental gene expression changes in cultured neonatal rat ventricular myocytes [29]. Furthermore, treatment with serum from heart failure (HF) patients or acute myocardial infraction (MI) has been shown to induce apoptosis and enhanced sprouting angiogenesis of cultured endothelial cells [30,31]. We used a similar approach to mimic the cardiac cellular response to systemic inflammation in the course of MCD. By the exposure of human cardiomyocytes and cardiofibroblasts to serum from acute MCD patients, we established the effects of this treatment on complex changes in the gene expression (small-scale RNA sequencing experiment). Based on clinical premises and observations from our initial RNA sequencing study, we also performed larger-scale experiment to further investigate the effects of MCD serum treatment on the genes associated with cellular iron homeostasis.

Our initial transcriptomic study showed that hCM and hCF treated with acute MCD patients’ sera exhibit the pathological changes in the gene expression, as compared with treatment with sera from healthy controls. Interestingly, very few genes were up- or downregulated in both cell lines simultaneously, suggesting unique cell-specific response in both cell types. Canonical pathway analysis revealed disturbances in the expression of genes involved in inflammation, endocytosis, virus entry via endocytic pathways, and apoptosis in both cell lines. Importantly, exclusively in hCM, we noticed a significant change in the iron homeostasis signaling pathway. Whereas, in hCF, a dysregulation of pathways involved in acute phase response was present. Additionally, observed alterations in the pathways such as HIPPO signaling, iNOS signaling, or Myc-mediated apoptosis are particularly interesting and have been shown to be involved in the pathophysiology of different heart diseases [32,33,34,35]. Interestingly, iron availability has been shown to be a significant modulator of HIPPO [36], iNOS [37], and Myc-mediated [38] signaling pathways, justifying our special interest in iron homeostasis. Moreover, both acute MCD sera-treated cell lines exhibited significantly dysregulated cardiotoxicity functions defined by IPA such as involvement in cardiac enlargement, cardiac dilation, cardiac inflammation, and heart failure. At this point, it is important to acknowledge that the unadjusted *p*-value (*p* < 0.05) used in our pathway analysis may result in false positives. However, the goal of this transcriptomic experiment was to identify the changes in a larger subset of genes to get a more complex view and to generate hypotheses on the alterations in pathways that are relevant in the course of myocarditis. Even after FDR correction, we still have found 38 and 8 significantly different genes in hCM and hCF, respectively. We have not found any particular relation between them; however, some of them might play a role in processes such as protein synthesis regulation in cardiomyocytes (PABPC1 [39]), cardiac differentiation and development (FURIN [40]; ADAR [41]), or cellular response to viral infection (TMED2 [42]). Complex response of both cell lines to the acute MCD serum treatment, especially in the context of disturbed canonical pathways and cardiac disease-related genes, suggest our model as efficient way to investigate the effects of systemic inflammation on cardiac cells in the course of MCD.

To further investigate the role of dysregulated iron homeostasis in course of MCD, we performed a larger-scale experiment where we treated hCM and hCF with sera from 18 MCD patients collected in two timepoints (acute phase and after 6 weeks of clinical recovery) and compared with 10 healthy controls. In support of our clinical observations and transcriptomic evidence for alterations in the iron homeostasis signaling pathway, we demonstrated that the expression of transferrin receptor 1 (TFR1) in both cell lines treated with MCD patient’s serum is increased. Ubiquitously expressed TFR1 is a membrane glycoprotein that serves as a gatekeeper in regulating the cellular uptake of iron from transferrin, and its overexpression is correlated with higher iron demand in cells [24,43]. In our previous studies on intracellular iron deficiency, we implied that TFR1 is upregulated in cardiomyocytes cultured in iron-deficient conditions and strongly correlates with decreased intracellular iron concentration [22]. Furthermore, the upregulated expression of TFR1 is associated with impaired cardiomyocytes viability and increased apoptosis [22,23]. Finally, due to an important role of iron in energy metabolism as a cofactor of four of five mitochondrial complexes, iron deficiency has been shown to directly affect cardiomyocyte function, impairing mitochondrial respiration and reducing contractility and relaxation [9]. At the tissue level, numerous studies have shown that iron deficiency (ID) in cardiac muscle results in cardiomegaly, left ventricular (LV) dilation, LV hypertrophy, cardiac fibrosis, and symptomatic HF [7,21,44,45]. The correlation between increased expression of TFR1 in our in vitro model and the diagnostic parameters of iron status of patients whose sera were used for treatment indicates that cells adapt to patient-specific iron availability and confirms the rationale of this in vitro model. Moreover, an increase observed in TFR1 expression was also observed in hCM and hCF treated with patients’ sera collected after 6 weeks of clinical recovery, showing that the iron-depletive effect of serum-treatment is continuous and persists at least several weeks after acute phase of myocarditis.

Moreover, we measured the expression of intracellular iron storage protein i.e., ferritin. However, we only observed an increase at the mRNA level in cells treated with acute MCD patient’s serum, without any effect at the protein level. This is partially in agreement with our observation of highly elevated serum ferritin in patients during the acute phase of MCD. At this point, it is important to emphasize that the assessment of iron status during concomitant inflammation is difficult because of its confounding effects on the interpretation of iron indicators [46]. Broadly used ferritin, which is a positive acute-phase protein, is highly elevated during states of inflammation, which is most likely in response to increasing amounts of cytokines [47]. Thus, the increase of ferritin mRNA in MCD-sera treated hCM and hCF may be rather related to its role as the acute phase protein than the increase of iron storage. Additionally, a lack of changes in ferritin protein expression (both cellular and extracellular) might be due to relatively short time of serum treatment (48 h)—it may be enough to induce the gene expression but not enough to increase the expression of protein.

Finally, in both cell lines treated with sera from MCD patients, we observed that the increased iron demand reflected by upregulated expression of TFR1 correlates with higher markers of systemic inflammation (CRP, IL-6) and advanced neurohormonal activation (NT-proBNP). Notably, extra and intracellular iron homeostasis is intimately tied to the inflammatory response and has been elucidated in numerous review studies [48,49]. Acute phase proteins such as ferritin, transferrin, haptoglobin, and hepcidin are activated by the acute phase response and may affect the distribution of iron to cells [48]. Additionally, inflammation can also affect the iron status by the reduction of intestinal absorption [50]. Therefore, observed changes in iron homeostasis seem to be important in the pathophysiology of MCD, while the major pathomechanisms of MCD and development of post-myocarditis non-ischemic CM are based on the action of immune cells, cardiomyocytes and cardiofibroblasts, which are very sensitive for changes in iron status.

### Study Limitations

The control group was slightly older than MCD patients. However, we did not observe any significant age-related associations in all measured parameters. Any age-related effects on systemic inflammation were not expected due to this relatively small difference in age [51]. Furthermore, we examined 18 consecutive patients with MCD, which were men only, and there are no data regarding women. However, it is worth emphasizing that a predominancy of male sex is a known feature of MCD [52,53].

## 5. Conclusions

This study showed that the treatment of primary cardiomyocytes and cardiofibroblasts with sera from MCD patients may represent an effective model for future studies on the mechanisms involved in this disease. Our results suggest that systemic inflammation occurring in the course of myocarditis results in the alterations in the cardiac iron homeostasis and may be involved in the pathological changes in cardiac cells. Further investigation on the molecular mechanisms of iron metabolism in MCD might reveal efficient therapeutic options to prevent its progression to inflammatory cardiomyopathy.

## Figures and Tables

**Table 1:** Primers and sequences used for qPCR.

Gene	Reference	
*TfR1*	qHsaCID0022106 (Bio-Rad PrimePCR™)	
*UBC*	ge-SY-12 (PrimerDesign)	
*ATP5B*	ge-SY-12; PrimerDesign	
*RQ1/RQ2*	qHsaCtlD0001002 (Bio-Rad PrimePCR™)	
**Gene**	**Sequence forward (5′–3′)**	**Sequence reverse (5′–3′)**
*FTH*	GCTCTACGCCTCCTACGTTT	GAAGATTCGGCCACCTCGTT
*FTL*	ATTTCGACCGCGATGATGTG	CATGGCGTCTGGGGTTTTAC

**Table 2:** Antibodies and dilutions used for Western blotting.

Antigen	Dilution	Manufacturer	Ref. Number
TFR1	1:500	Abcam	ab84036
FTH	1:500	Abcam	ab81444
FTL	1:5000	Abcam	ab186871
Actin HRP	1:5000	Santa Cruz Biotechnology	sc-1616 HRP
Rabbit IgG HRP	1:40,000	Jackson ImmunoResearch	111-035-045

**Table 3:** Baseline characteristics of acute myocarditis patients and healthy controls. Data are presented as a mean ± standard deviation of the mean, a median (with an interquartile range), where appropriate. Abbreviations: N, number of patients for whom the parameter was available; LVEF, left ventricular ejection fraction; CRP, C-reactive protein; ALT, alanine transaminase; NT-proBNP, N-terminal pro-B-type natriuretic peptide; IL-6, interleukin 6; Student’ *t*-test or Mann–Whitney test (depending on data normaility).* *p* < 0.05; ** *p* < 0.01; *** *p* < 0.001.

Parameter	N	Healthy Controls	N	Myocarditis (Acute Phase)	Acute Phase vs. Healthy *p*-Value	N	Myocarditis (After 6 Weeks of Clinical Recovery)
Age (years)	10	46 ± 13	18	33 ± 8.8	0.005	18	33 ± 8.8
Sex (% Male)	10	100%	18	100%	-	18	100%
LVEF (%)	10	65 ± 4.7	18	58 ± 9.4	0.027	17	58 ± 7.8
CRP (mg/L)	10	0.63(0.43–1.50)	18	17(6.8–63.0)	<0.0001	17	1(0.6–3.8)
ALT (IU/L)	10	29(21–37)	18	30(25–55)	0.23	17	22(17–39)
Troponin	10	0.01 (0.01–0.01)	18	0.87(0.26–5.7)	<0.0001	17	0.01(0.01–0.01)
Serum creatinine (mg/dL)	10	0.88(0.86–0.97)		0.94(0.82–1.00)	0.99	17	0.90(0.82–0.98)
NT-proBNP (pg/mL)	10	40(16–77)	18	315(98–939)	0.0005	16	32(26–62)
IL-6 (pg/mL)	10	0.73(0.2–1.9)	16	4.5(1.7–7.9)	0.0003	17	0.69(0.1–0.69)
**Hematological Parameters and Indices of Iron Status**							
Hemoglobin concentration (g/dL)	10	15.2 ± 1.2	18	15 ± 1.1	0.29	17	15 ± 0.94
Serum iron (μg/dL)	10	102 ± 47	18	69 ± 29	0.029	16	88 ± 20
Serum ferritin (μg/L)	10	154(78–213)	18	234(150–418)	0.026	16	160(112–187)
Soluble transferrin receptor (mg/L)	8	1.2(1.0–1.6)	18	1.2(1.0–1.4)	0.81	17	1.2(1.1–1.3)
Transferrin saturation (%)	10	30 ± 13	18	21 ± 8.4	0.052	17	25 ± 5.3

## Data Availability

The data presented in this study are available on request from the corresponding author. The data are not publicly available due to personal data protection.

## Supplementary Materials

The following are available online at https://www.mdpi.com/article/10.3390/cells10040818/s1, Table S1: IPA data analysis: Alterations in Ingenuity Canonical Pathways in cells treated with sera from healthy controls vs. myocarditis patients. Figure S1: IPA data analysis: Alterations in disease-related pathways in cells treated with sera from healthy controls vs. myocarditis patients.
