# Supplementary material for: Primary Human Cardiomyocytes and Cardiofibroblasts Treated with Sera from Myocarditis Patients Exhibit an Increased Iron Demand and Complex Changes in the Gene Expression
Source: Cells. 2021 Apr 6;10(4):818. doi: 10.3390/cells10040818 (PMC8067399; doi:10.3390/cells10040818)

Supplementary Table 1. IPA data analysis: Alterations in Ingenuity Canonical Pathways in cells treated with sera from healthy controls vs. myocarditis patients

A) hCM

**Pathway**	**-log(p-value)**
Huntington's Disease Signaling	4.475
Salvage Pathways of Pyrimidine Ribonucleotides	3.052
Virus Entry via Endocytic Pathways	2.575
HIPPO Signaling	2.513
Cholesterol biosythesis I	2.507
Cholesterol Biosynthesis II (via 24,25-dihydrolanosterol)	2.507
Cholesterol Biosynthesis III (via Desmosterol)	2.507
Myc Mediated Apoptosis Signaling	2.31
ERK5 Signaling	2.019
Superpathway of Cholesterol Biosynthesis	1.932
iNOS Signaling	1.666
Iron homeostasis signaling pathway	1.5
Renal Cell Carcinoma Signaling	2.226
Pyridoxal 5'-phosphate Salvage Pathway	2.356
Hupusine Biosynthesis	2.139
Aryl Hydrocarbon Receptor Signaling	1.697
Serine Biosynthesis	1.646
Tetrapyrrole Biosynthesis II	1.646
Caveolar-mediated Endocytosis Signaling	1.535
Estrogen-Dependent Breast Cancer Signaling	1.52

B) hCF

**Pathway**	**-log(p-value)**
Clathrin-mediated Endocytosis Signaling	4.07
PAK Signaling	3.33
Myc Mediated Apoptosis Signaling	3.12
Molecular Mechanisms of Cancer	3
Paxillin Signaling	2.91
Rac Signaling	2.73
Signaling by Rho Family GTPases	2.71
14-3-3-mediated Signaling	2.68
PTEN Signaling	2.68
Breast Cancer Regulation by Stathmin1	2.64
CTLA4 Signaling in Cytotoxic T Lymphocytes	2.61

Agrin Interactions at Neuromuscular Junction	2.59
Acute Phase Response Signaling	2.42
Virus Entry via Endocytic Pathways	2.41
Sertoli Cell-Sertoli Cell Junction Signaling	2.35
VEGF Signaling	2.28
Mismatch Repair in Eukaryotes	2.27
HER-2 Signaling in Breast Cancer	2.27
Ephrin A Signaling	2.26
NGF Signaling	2.21
Apelin Liver Signaling Pathway	2.21
CD28 Signaling in T Helper Cells	2.15
B Cell Receptor Signaling	2.02
Germ Cell-Sertoli Cell Junction Signaling	1.99
Hereditary Breast Cancer Signaling	1.95
Role of BRCA1 in DNA Damage Response	1.94
Neuregulin Signaling	1.94
Molybdenum Cofactor Biosynthesis	1.92
HGF Signaling	1.89
Endocannabinoid Cancer Inhibition Pathway	1.83
Actin Nucleation by ARP-WASP Complex	1.82
GM-CSF Signaling	1.82
Reelin Signaling in Neurons	1.8
Role of CHK Proteins in Cell Cycle Checkpoint Control	1.8
3-phosphoinositide Biosynthesis	1.8
BER pathway	1.8
Superpathway of Inositol Phosphate Compounds	1.79
ERK5 Signaling	1.75
IL-7 Signaling Pathway	1.71
STAT3 Pathway	1.64
RhoA Signaling	1.61
IL-6 Signaling	1.52

Supplementary Figure 1. IPA data analysis: Alterations in disease-related pathways in cells treated with sera from healthy controls vs. myocarditis patients

A) hCM

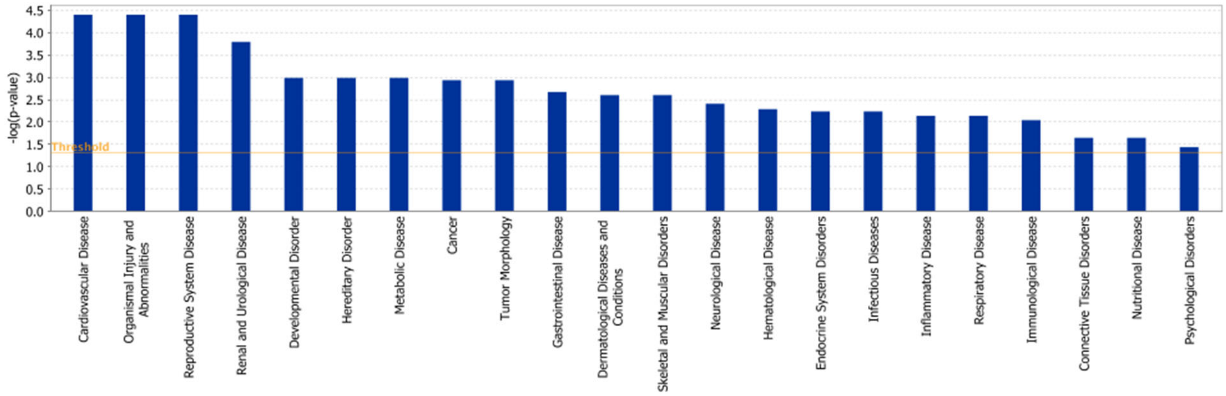

B) hCF

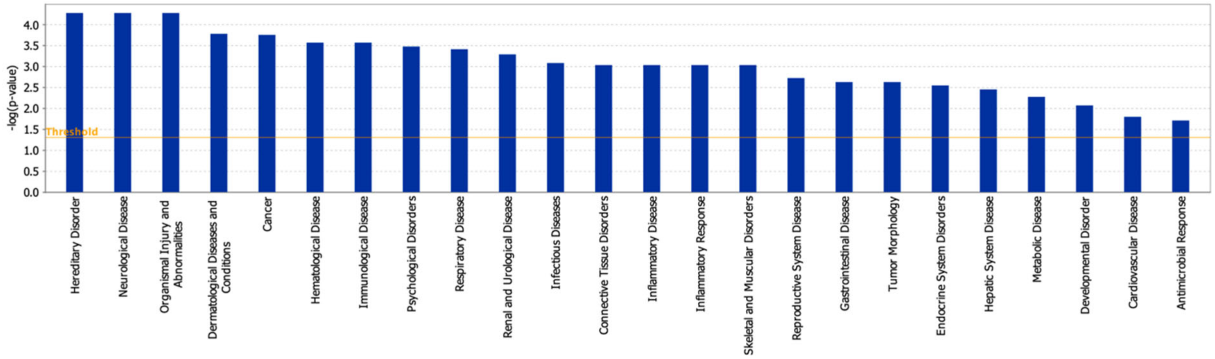

Supplement: Supplementary file 1 [file cells-10-00818-s001.pdf]
